# Coping with COVID-19 in an international border region: health and economy*

**DOI:** 10.1590/1518-8345.4659.3398

**Published:** 2021-01-08

**Authors:** Reinaldo Antonio Silva-Sobrinho, Adriana Zilly, Rosane Meire Munhak da Silva, Marcos Augusto Moraes Arcoverde, Enrique Jorge Deschutter, Pedro Fredemir Palha, Angela Sobral Bernardi

**Affiliations:** 1 Universidade Estadual do Oeste do Paraná, Campus de Foz do Iguaçu, Foz do Iguaçu, PR, Brazil.; 2 Universidad Nacional de Misiones, Posadas, MI, Argentina.; 3 Universidade de São Paulo, Escola de Enfermagem de Ribeirão Preto, PAHO/WHO Collaborating Centre for Nursing Research Development, Ribeirão Preto, SP, Brazil.

**Keywords:** Pandemics, Covid-19, Social Isolation, Border Areas, Public Health, Border Health, Pandemias, Covid-19, Isolamento Social, Áreas de Fronteira, Saúde Pública, Saúde na Fronteira, Pandemias, Covid-19, Aislamiento Social, Áreas Fronterizas, Salud Pública, Salud Fronteriza

## Abstract

**Objective:**

to analyze how the social isolation measures and closed borders affected the health and economy in an international border region.

**Method:**

descriptive cross-sectional study conducted in the western region of Paraná, Brazil, using an electronic form created using Google^®^ forms. A sample of 2,510 people was addressed. Descriptive analysis and the Chi-square test were performed, with a level of significance established at 5%. This public opinion survey, addressing unidentified participants, is in accordance with Resolutions 466/2012 and 510/2016.

**Results:**

the participants were 41.5 years old on average, most were women and worked in the education sector; 41.9% reported that the closing of borders/commercial businesses negatively influenced income; 17.7% reported the possibility of losing their jobs; 89.0% consider that a larger number of people would be sick if the borders/commercial had not been closed; 63.7% believe the health services are not prepared to deal with the pandemic; 74.9% realize that the Brazilian Unified Health System may not have sufficient service capacity; 63.4% reported anxiety; and 75.6% of commercial workers will experience changes in their income level.

**Conclusion:**

the closing of international borders and commercial businesses was related to a perception of physical and mental changes, job loss, and decreased income.

## Abstract (pt)

**Objetivo:**

analisar como o isolamento social e o fechamento das fronteiras repercutem na saúde e na economia em região de fronteira internacional.

**Método:**

estudo descritivo-transversal realizado no Oeste do Paraná, Brasil, por meio do questionário eletrônico Formulários Google^®^. Foi estudada uma amostra de 2.510 pessoas. Utilizou-se a análise estatística descritiva e o teste qui-quadrado, com nível de significância de 5%. Pesquisa de opinião pública, com participantes não identificados, que atende às Resoluções 466/2012 e 510/2016.

**Resultados:**

a média de idade foi de 41,5 anos, a maioria é do sexo feminino e composta por trabalhadores do setor de educação; 41,9% indicam que o fechamento das fronteiras/comércio influenciou negativamente a renda e, para 17,7%, existe a possibilidade de desemprego. Para 89,0%, o número de pessoas adoecidas seria maior caso as fronteiras/comércio não tivessem sido fechadas; 63,7% indicam que os serviços de saúde não estão preparados para enfrentar a pandemia; 74,9% percebem que o Sistema Único de Saúde pode não ter capacidade de atendimento; 63,4% sinalizam ansiedade e 75,6% dos trabalhadores do comércio terão alterações na renda.

**Conclusão:**

o fechamento das fronteiras internacionais e do comércio relacionou-se à percepção de alterações físicas e mentais, perda de emprego e de renda.

## Abstract (es)

**Objetivo:**

analizar cómo el aislamiento social y el cierre de las fronteras afectan la salud y la economía en una región fronteriza internacional.

**Método:**

estudio descriptivo transversal desarrollado en el occidente de Paraná, Brasil, utilizando el cuestionario electrónico Formularios Google^®^. Se estudió una muestra de 2.510 personas. Se utilizó análisis estadístico descriptivo, prueba de chi-cuadrado con nivel de significancia del 5%. Encuesta de opinión pública, con participantes no identificados, que cumple con las Resoluciones 466/2012 y 510/2016.

**Resultados:**

el promedio de edad fue de 41,5 años, mayoritariamente mujeres y trabajadores del sector educativo; el 41,9% indicó que el cierre de las fronteras/comercio afectó negativamente los ingresos y que, para el 17,7%, existe la posibilidad de desempleo. Para 89,0%, el número de personas enfermas habría sido mayor si las fronteras/comercio no se hubieran cerrado; 63,7% indica que los servicios de salud no están preparados para enfrentar la pandemia; 74,9% percibe que el Sistema Único de Salud puede no ser capaz de brindar atención; 63,4% informó ansiedad; y 75,6% de los trabajadores del comercio tendrán cambios en los ingresos.

**Conclusión:**

el cierre de las fronteras internacionales y el comercio se relacionó con la percepción de cambios físicos y mentales, pérdida de empleo e ingresos.

## Introduction

COVID-19 is an acute respiratory disease that presents hematological changes caused by the novel Severe Acute Respiratory Syndrome Coronavirus 2 (SARS-CoV-2) and presents a mortality rate ranging from 0.5% to 18%, according to age^(^ 1 ^)^. It was first described in December 2019, in Wuhan, China, and spread over all the continents^(^ 2 ^)^.

It is a rapidly evolving disease due to alveolar damage and progressive respiratory failure, which requires immediate ventilation support^(^ 3 ^)^. The main signs and symptoms include dyspnea, dry cough, fever, body pain, and sore throat, runny nose, and skin rashes^(^ 3 ^-^ 4 ^)^.

More than 6.7 million people were infected with COVID-19 worldwide. Even though the largest portion of cases is concentrated in the United States of America (USA), with almost 2 million people infected, the virus has affected all continents, especially Russia, Spain, the United Kingdom, Italy, Germany, Brazil, Turkey, and France. Deaths have surpassed 390,000 people, with alarming proportions in many countries^(^ 1 ^)^.

The disease’s spread in Brazil follows the same growing speed, with variations depending on the regions, states, and cities. There was a significant increase in the number of cases and deaths, despite social isolation measures intended to contain the virus^(^ 1 ^,^ 5 ^)^, revealing the current health context is of concern. Brazil exceeded 2 million cases up to July 2020, and lethality reached 3.8% with an incidence of 1,031.8 cases/100,000 inhabitants and 38.8 deaths/100,000 inhabitants. There is potential underreporting, though, considering there is a lack of tests for the Brazilian population. Thus, the number of cases confirmed thus far is expected to be six times higher^(^ 1 ^)^.

A systematic review reports that because there is no vaccine or medication with robust *in vivo* scientific evidence thus far, social isolation is the primary strategy to decrease the number of new cases and deaths^(^ 6 ^)^ in the population in general. Scientific evidence confirms that social isolation is efficient; however, it also decreases economic activity in general^(^ 5 ^)^. A combination of social isolation and the release of essential activities only, associated with individual preventive and control measures, such as the use of face masks and alcohol at 70%, present a more significant effect^(^ 5 ^-^ 6 ^)^.

If, on the one hand, social isolation practices have been proven to be a good measure to contain the spread of COVID-19, on the other hand, it directly affects the economy at a global and local level. It is important to note that decreased industry and commercial activities and services, impact the businesses’ health. The devastating effect of such measures on economic activities has to be taken into account; however, resuming the economic activity too soon and without proper monitoring may cause the inverse of the expected effect, that is, general mortality rates may rise while the economy’s ability to recover and time for it to recover may decrease^(^ 7 ^)^.

Border cities, which compose this study’s setting, are facing a dilemma between control and integration. Access and circulation of people result from labor relationships, tourism, consumption, and the need to use public health and education services^(^ 8 ^)^. Brazilians, Paraguayans, and Argentineans cross the border every day to access different services, be it for trading, industrial or labor relations, access formal education, use health services or religious practices, and tourism, among others. In this context, both the health and economic sectors are affected by the COVID-19 pandemic.

Because the region under study, more specifically the city of Foz do Iguaçu, Paraná, Brazil, has many natural and human-made attractions, tourism figures among its main economic activities^(^ 8 ^)^. Some indicators show the relevance of the city and this border region. It is considered the second most frequently visited destination by international tourists and the largest free zone in Latin America. In 2019, Iguaçu Falls received more than 2 million visitors from 177 countries, and the Itaipu Binacional hydroelectric Complex was visited by more than 1 million national and international tourists^(^ 7 ^)^. Hence, the repercussions on the economy and population’s health are apparent, given borders closure and the adoption of social isolation measures in the region.

This context will lead to direct and indirect consequences such as job losses and salary reductions, directly impacting the families’ income. One of the current main challenges is to find alternatives, besides the emergence actions of the federal government, to keep the income of families, considering the significant dependence on tourism and commercial relations between cross-border countries^(^ 7 ^)^, without, however, worsening the area’s sanitary and economic conditions^(^ 5 ^-^ 6 ^)^. This study’s objective was to analyze the repercussions of social isolation and border closure on the health and economic conditions of the population living in an international border region.

## Method

This descriptive cross-sectional study was conducted in the cities of the Western Paraná, Brazil, with a population estimated in 1,219,548 inhabitants, according to the 2010 Census. The region has 50 cities, 17 of which border Paraguay and Argentina. The region’s main economic vocations that generate employment and income are agriculture, tourism, and the transportation and trade of goods coming from the MERCOSUR. The region has 284 primary health care units, 32 public hospitals with 2,039 beds designated for patients depending on the Brazilian Unified Health System (SUS), and 188 beds in Intensive Care Units (ICUs), also designated for SUS. Note that these facilities also receive patients coming from Paraguay and Argentina (i.e., both international and Brazilian emigrants)^(^ 9 ^)^.

Data were collected from April 10^th^ to 13^th^, 2020. The participants were volunteers, formal or informal workers, aged over 18 years old, living in the cities located in the western region of Paraná. Those who did not report their age or their city of residence were excluded. A total of 2,697 individuals answered the questionnaire, 2,510 of whom met the inclusion criteria.

The sample size calculation took into account the population living in the cities included in this study, an error (∊) of 2%, and a 95% confidence interval (Z). A sample size of 2,397 participants resulted from the formula:

$$\mathrm{Sample}\;\mathrm{size}=\frac{\frac{z^{2}\times p\left(1-p\right)}{e^{2}}}{1+\left(\frac{z^{2}\times p\left(1-p\right)}{e^{2}N}\right)}$$

A total of 2,510 participants answered the electronic questionnaire and met the inclusion criteria.

A questionnaire was developed to collect opinions regarding the impact of social isolation measures and the closing of international borders on health and the region’s economy. This structured questionnaire was assessed by three experts in the field of collective health. It is a 32-item form with three open-ended questions, six multiple-choice questions, and 23 dichotomic questions (yes or no), comprising five dimensions: 1) Sociodemographic data; 2) Economic impact; 3) Sanitary measures to contain the pandemic; 4) The responses of the city’s public services to deal with the pandemic, and 5) The impact of social isolation on health.

Data were collected using an electronic questionnaire developed with Google Forms^®^, directed to the population living in the cities under study. The questionnaire was available online, disseminated through social media and on the official website of the Western Paraná State University.

Descriptive statistics were used, presenting absolute and relative frequencies. Association between variables was verified using the Chi-square test and a 5% significance level. Association between the answers provided by the different groups of workers was verified to develop the contingency tables. Data were tabulated in spreadsheets in the Microsoft Office Excel 2016 and analyzed using R, version 3.6.1.

This public opinion survey addressed unidentified respondents and is in accordance with the Resolutions 466/2012 and 510/2016 established by the Brazilian National Health Council, Ministry of Health.

## Results

The participants were 41.5 years old on average (SD±12.3) and had 1.4 children on average (SD±1.4).

Most were women (67.7%), married (57.1%), with children (69.4%), worked in the education sector, in public or private schools (19.6%), with statistically significant differences between professions (p<0.001), as shown in Table 1.

**Table 1:** Distribution of the participants' sociodemographic variables. International border region of Western Paraná, Brazil, 2020

Variables (n=2,510)	N	%	P-value
Sex			<0.001
Women	1700	67.7	
Men	786	31.3	
Others	4	0.2	
Did not answer	20	0.8	
Marital Status			<0.001
Married	1285	57.1	
Single	608	27.0	
Stable union	290	13.0	
Widowed	37	1.6	
Other	30	1.3	
Children			<0.001
Yes	1743	69.4	
No	767	30.6	
Employment Sector			<0.001
Commerce	324	12.9	
Homemaker	130	5.2	
Education (public/private)	492	19.6	
Student	141	5.6	
Public employee	431	17.2	
Health	457	18.2	
Tourism	441	3.1	
Other	78	17.6	
Did not answer	16	0.6	

Regarding housing expenditure, 55.1% (n=1,382) reported they did not pay for rent; 13.1% (n=330) reported the possibility of not having enough money to pay for the rent during the months of social isolation and border closure. As for expenditure with healthcare services, 38.4% (n=384) reported they did not pay private health insurance/plan, and 35% (n=879) reported they depended on SUS. Note that due to the border closure, 13.7% reported that they may not have enough money to pay for their health insurance/plan, in which case, they will migrate from the private health system to become exclusively dependent on SUS. Another factor for concern is that 14.8% (n=371) report they may not have enough money to buy medication during the period. A p<0.001 was found for both the analysis involving housing and health (SUS and medication) expenditures.

Given the repercussions of sanitary measures on the economy, the respondents consider that the closure of the international borders influences or will influence their family income (41.9%) (Table 2). This percentage increases to 58.5% when the question specifically refers to the respondent’s city of residence (p<0.001).

**Table 2:** Distribution of the variables regarding the economic repercussions of sanitary measures to deal with the COVID-19 pandemic. International border region of Western Paraná, Brazil, 2020

Variables (n=2,510)	n	%	P-value
Did the border closure, due to the COVID-19 pandemic, affect, or will affect your family income?			<0.001
No	1452	57.9	
Yes	1052	41.9	
Did not answer	6	0.2	
Will you have enough money this month to buy food?			<0.001
No	2120	84.5	
Yes	385	15.3	
Did not answer	5	0.2	
Will you have enough money to pay water, electricity, telephone and/or internet bills?			<0.001
No	1876	74.8	
Yes	631	25.1	
Did not answer	3	0.1	
Are you at the risk of losing your job because of social isolation (commercial business closure, stay-at-home orders)?			<0.001
No	1782	71.0	
Yes	446	17.8	
I was unemployed before social isolation	205	8.1	
I have already lost my job because of social isolation	65	2.6	
Did not answer	12	0.5	
Tourism and economic activities currently affected by social isolation will continue to be affected even after the border and commercial are opened?			<0.001
No	123	4.9	
Yes	2380	94.8	
Did not answer	7	0.3	

The respondents were also asked whether they might not have money for essentials, and there were affirmative answers regarding the possibility of not having money to buy food (15.3%), or pay water, electricity, telephone and/or internet bills (25.1%) (Table 2). A total of 17.8% of the respondents report the possibility of becoming unemployed while 2.6% reported they had already lost their jobs with the closing of borders and of commercial businesses and social isolation, with p<0.001 (Table 2).

Regarding the need to close international borders, commercial businesses, and schools/universities to prevent the spread of SARS/CoV2/COVID-19, 90.8%, 79.8%, and 91.5% (p<0.001), respectively agreed these were necessary measures. A total of 89.0% consider that if the border and commercial businesses had not been closed, the number of individuals infected by SARS/CoV2 would have been even higher (p<0.001), as shown in Table 3.

**Table 3:** Distribution of the variables regarding sanitary measures and responses of public services to deal with the COVID-19. International border region of Western Paraná, Brazil, 2020

Variables (n=2.510)	N	%	P
Is border closure a useful measure to prevent people from becoming sick with COVID-19?			<0.001
No	226	9.0	
Yes	2279	90.8	
Did not answer	5	0.2	
Is the closing of commercial businesses in your city a useful measure to prevent people from becoming sick with COVID-19?			<0.001
No	496	19.8	
Yes	2003	79.8	
Did not answer	11	0.4	
Is the closing of schools and universities a useful measure to prevent people from becoming sick with COVID-19?			<0.001
No	200	8.00	
Yes	2298	91.5	
Did not answer	12	0.5	
Are the health services prepared to provide care to the population in the context of the COVID-19 pandemic?			<0.001
No	1599	63.7	
Yes	901	35.9	
Did not answer	10	0.4	
Are the public health services (SUS) still providing care to the population regarding other health problems (for instance, dengue, hypertension, and urgent/emergence care, among others)?			<0.001
No	915	36.4	
Yes	1583	63.1	
Did not answer	12	0.5	
Is your city at the risk of losing the capacity to provide care to people with symptoms and/or infected with COVID-19?			<0.001
No	614	24.5	
Yes	1881	74.9	
Did not answer	15	0.6	

Table 3 also shows that 63.7% consider that the health services are not prepared to provide care to individuals with SARS/CoV2/COVID-19, and 74.9% consider the SUS may not have the capacity to provide care to individuals with symptoms and/or infected by the virus (p<0.001).

Another piece of data analyzed was the population’s perception regarding the governments’ responses (at the municipal, state, and federal levels) to deal with the COVID-19 pandemic. In this context, 70.3% (n=1,764) agree that the government, at all levels, acted rapidly and made decisions to care for the population’s health (p<0.001).

In the questions assessing the impact of social isolation, Table 4 shows that 63.5% of the respondents reported that social isolation caused anxiety, and 32.3% related the onset of body pain they had not previously experienced to social isolation (p<0.001).

**Table 4:** Distribution of variables regarding the impact of social isolation on health due to the COVID-19. International border region of Western Paraná, Brazil, 2020

Variables (n=2,510)	n	%	P-value
Did social isolation affect your mood?			<0.001
No	1137	45.3	
Yes	1365	54.4	
Did not answer	8	0.3	
Did social isolation cause the emergence of body pain you had not previously experienced?			<0.001
No	1692	67.4	
Yes	811	32.3	
Did not answer	7	0.3	
Did social isolation change your mental state, causing sadness?			<0.001
No	1326	52.8	
Yes	1176	46.9	
Did not answer	8	0.3	
Did social isolation change your mental state, causing anxiety?			<0.001
No	906	36.1	
Yes	1593	63.5	
Did not answer	11	0.4	
Would the number of individuals infected with the COVID-19 be greater if the borders and commercial businesses had not been closed?			<0.001
No	242	9.6	
Yes	2254	89.8	
Did not answer	14	0.6	

Additionally, 7.8% of the respondents reported that social isolation caused the emergence of diseases they had not previously experienced (p<0.001).

The results show that workers from different sectors report that border closure will influence their family income, especially those in commercial/tourism businesses (75.6%; p<0.001). Even workers with some job stability reported income losses (Table 5).

**Table 5:** Distribution of the variables economic impact, sanitary measures, and responses of the public health services to deal with the pandemic according to different professions. International border region of Western Paraná, Brazil, 2020

Variables	Commerce/tourism		Public servers/ Education ^†^		Health workers^‡^		Others^§^		P-value
	n	%	N	%	n	%	n	%	
Did the border closure impact your family income? (n=2,488)									<0.001
Yes	303	75.6	273	29.6	169	37.0	298	42.0	
No	98	24.4	648	70.4	288	63.0	411	58.0	
Border closure is a useful measure to prevent people from becoming sick? (n=2.489)									<0.001
Yes	327	81.5	881	95.7	423	92.6	633	89.2	
No	74	18.5	40	4.3	34	7.4	77	10.8	
Are the health services prepared to provide care to the population in the context of the coronavirus? (n =2,484)									0.261
Yes	159	39.7	314	34.2	171	37.4	252	64.5	
No	242	60.3	603	65.8	286	62.6	457	35.5	
Is the SUS in your city at the risk of losing its capacity to care for people with symptoms and/or infected with the COVID-19? (n=2,479)									<0.001
No	113	28.2	181	19.7	117	25.7	198	28.1	
Yes	288	71.8	736	80.3	339	74.3	507	71.9	
Social isolation caused the onset of body pain you have not experienced before? (n=2,487)									<0.001
No	237	59.1	655	71.2	308	67.5	481	67.7	
Yes	164	40.9	265	28.8	148	32.5	229	32.3	
Did social isolation change your mental state, causing anxiety? (n=2,483)									<0.001
No	105	26.4	368	40.0	164	36.0	263	37.1	
Yes	293	73.6	552	60.0	292	64.0	446	62.9	
Would the number of individuals infected by the coronavirus be higher if the border and commercial businesses had not been closed? (n=2,480)									<0.001
No	77	19.3	44	4.8	33	7.2	85	12.0	
Yes	321	80.7	875	95.2	424	92.8	621	88.0	

* Commerce/tourism = Workers from the trade/tourism sectors;

† Public servers/Education = Public employees and workers from the education sector (private and public);

‡ Health workers = Health workers from the public or private sectors;

§ Others = Homemakers, students, other occupations

Analysis of the impact of closing commercial businesses shows that workers in the commercial/tourism businesses and those in the *Others* category (i.e., homemakers, students, other occupations) were the most frequently affected, that is, 87% and 60.6%, respectively, reported that the measure influenced or will influence their families’ income (p<0.001).

The highest impact of the closure of commercial businesses was reported by those working in commercial/tourism businesses (87%) followed by 60.6% of those in the *others* category (i.e., homemakers, students, and other occupations) reported that this measure influenced or will influence their family income (p<0.001).

Whether the closing of borders was an appropriate measure to prevent the spread of SARS/CoV2/COVID-19, those in the commercial/tourism businesses more frequently disagreed, 18.5% (Table 5). This percentage virtually double folds (36.1%) when this question specifically refers to the closing of commercial businesses (p<0.001).

When asked whether the health services are prepared to provide care in the context of the COVID-19 pandemic, most of the respondents in the *Others* category answered affirmatively (64.5%). Note, however, the frequency of the negative answers *are not prepared* provided by health workers (62.6%) (though with no statistical significance) (Table 5).

When asked whether the SUS could lose its capacity to provide care to people with symptoms and infected with COVID-19, affirmative responses ranged from 71.8% (commerce/tourism workers) to 80.3% (education workers and public employees, p<0.001) (Table 5).

In the dimension addressing the impact of social isolation on health, 40.9% of workers from the commercial/tourism businesses reported that social isolation caused pain in their bodies they had never experienced before, showing the highest occurrence rate among the groups. Workers in the field of education and public employees; however, less frequently reported this impact (28.8%). For 73.6% of commercial/tourism workers and 60.0% of workers from the field of education and public employees, social isolation changed their mood, causing anxiety (p<0.001) (Table 5). Mood changes (p=0.002), sadness (p<0.001), and the onset of diseases (p<0.010) were especially reported by those working in the trade/tourism sectors. A total of 95.2% of those in the education field and public employees consider that the number of those infected with the COVID-19 would be higher if the international borders had not been closed. This percentage drops to 80.7% among those in the commercial/tourism businesses (p<0.001).

## Discussion

Social isolation has been an efficient measure to deal with the COVID-19 pandemic; however, this restrictive measure impacts both the economy and the health of the population, especially in border regions where income accrues from the commercial/tourism businesses and non-essential services. There are, in a border region, where commercial relations decrease due to the closing of borders, direct consequences on employment, income, lifestyle, and even the subsistence of families, affecting the working class and especially the most vulnerable groups^(^ 10 ^)^.

The repercussions of the COVID-19 pandemic in countries with a history of economic recession and low capacity to create and maintain job posts are even greater on the economy in general and on the lives of people. Even though developing countries are expected to face more difficulties in implementing social isolation measures, one should bear in mind that economy and health are not antagonistic aspects in times of sanitary crises. It is consensual that governments should assume the role of sanitary and economic authority to organize and promote access to food, hygiene goods, and health services^(^ 11 ^)^.

The economic impact caused by the COVID-19 will not be evenly distributed among the population in general, which will lead to increased social inequality within countries. Developed countries such as the USA verified income losses among workers and estimates are that unemployment will increase from 3.5% to 20%. These projections show that an increase in the number of unemployed individuals will be greater among already precarious activities with lower incomes^(^ 12 ^)^, with no prospects of recovery in the short-term.

These projections are consistent with this study’s findings. The need to implement social isolation measures, keeping open only those businesses that provide for essential activities, and the closing of international borders, resulted in immediate economic impact, causing the loss of jobs and decreased resources for families to meet their basic needs. As for sanitary measures to deal with the pandemic and the responses of public services^(^ 13 ^)^, in 2012, has been highlighted the direct relationship of rapid population displacement *versus* the emergence of epidemics and the need for health surveillance. Hence, the initiative of Paraguay and Argentina to close the Friendship bridge (between Brazil and Paraguay) and Fraternity bridge (between Brazil and Argentina) in March 2020, was important for controlling the spread of the virus, promoting social distancing, as recommended by the World Health Organization^(^ 14 ^-^ 15 ^)^.

Closing non-essential businesses and universities/schools in the borders was also a measure implemented by the three countries. In Brazil, it resulted from a decree enacted by the Paraná government^(^ 16 ^-^ 18 ^)^. These measures were intended to avoid agglomeration of people and contain the virus’ dissemination, decreasing and flattening the epidemic curve^(^ 19 ^)^. This study’s results show that the population understood the importance of these sanitary actions.

Of the nine hospitals designated to provide care to severe cases of COVID-19 in Paraná, two are located in the Western region, University hospital of Western Paraná and Padre Germano Lauck Municipal Hospital, located in Cascavel and Foz do Iguaçu, respectively. Ministro Costa Cavalcanti Hospital, in Foz do Iguaçu, maintained by the Itaipu Binacional, was also designated to care for severe cases and perform COVID-19 diagnostic tests^(^ 20 ^)^.

Most Brazilians (70%) exclusively depend on SUS and more than R$ 5 billion were designated to fight COVID-19^(^ 21 ^)^. However, Brazil presents different sanitary and epidemiologic profiles, and the way health services are distributed vary according to the socioeconomic development of each region, meaning SUS is confronted with considerable challenges to meet the population’s needs. For this reason, SUS is likely to become overburdened in some regions and cities, not being able to deal with both already existing health problems and those emerging because of the pandemic^(^ 22 ^)^.

Note that in addition to the COVID-19 pandemic, health services are concomitantly dealing with a dengue epidemic. For instance, Foz do Iguaçu, one of the cities addressed here, has more than 20,000 suspected cases, and the largest number of cases with autochthonous in the state (more than 6,000). Thus, in Paraná, SUS is dealing with the COVID-19 pandemic and dengue epidemic, in addition to scheduled visits and everyday spontaneous visits. Most respondents noticed this increased number of demands.

It is difficult to foresee all the consequences, considering that countries in Latin America, such as Brazil and its neighbors Paraguay and Argentina, are also dealing with basic health problems, problems other countries have already managed to control, such as dengue and tuberculosis, diseases that are associated with poverty and the health systems’ decreased response capacity^(^ 23 ^)^.

Regarding access to health services, only 35% of the sample reported they exclusively depended on SUS; the remaining had complementary health insurance/plans. Note, however, that a large number of respondents consider that SUS might not be able to meet the demand. It suggests that regardless of recognizing themselves as being currently exclusively dependent on SUS, the participants seem to understand that the health services’ installed capacity, whether SUS or not, may collapse due to the pandemic. Thus, all may indirectly become dependent on SUS, considering that private services may rapidly exhaust their installed capacity and families, in turn, may not be able to bear the costs of private health insurance/plans due to a decrease in income.

In this sense, the Brazilian Ministry of Health has, in partnership with state and city health departments, made investments to strengthen the SUS to face the pandemic. Actions include the National Contingency Plan for Human Infection with the New Coronavirus COVID-19, personnel hiring, the purchase of diagnostic tests, and the organization of hospital and primary health care facilities, among others. In the western region of Paraná, Brazil the pandemic required health services to be reorganized, expanding the number of ICU beds, buying equipment, and preparing laboratories to perform C-reactive protein (PCR) tests, in addition to the purchase of inputs. Note that this rapid response of SUS to deal with the COVID-19 does not only reach the SUS-dependent population, considering that access to SUS services is universal.

This study identified physical and psychological problems such as mood changes, anxiety, and pain. These results are in line with a study reporting that individuals experienced anxiety and depression during epidemics and pandemics, which are coupled with other changes in mental health^(^ 24 ^)^ that are related to social isolation and economic instability.

In extreme cases, distress may lead to suicidal behavior such as what happened among elderly individuals in 2003 in Hong Kong, during and after the Severe Acute Respiratory Syndrome pandemic^(^ 24 ^)^. There are current reports of suicides driven by the SARS/CoV2 pandemic in Bangladesh and India, mediated by prejudice and fear of disseminating the disease^(^ 25 ^-^ 26 ^)^. A factor to consider is that the educational level in these two countries is low, in which case, fear, coupled with a lack of information, may have led to such tragedies^(^ 25 ^)^.

For this reason, studies addressing mental health, social isolation, and the COVID-19 pandemic are essential to support strategies intended to improve the population’s mental wellbeing. Additionally, reliable information needs to be provided to minimize fear and panic and assist individuals with previous mental disorders, including telehealth services^(^ 27 ^)^.

Regarding the onset of physical symptoms, as verified in this study, it is essential to consider that stress, fear, intense worry are relevant in the etiology of many diseases given the activation mechanism of the sympathetic-adrenal-medullary system and the hypothalamic-pituitary-adrenal axis, which respectively release catecholamines and glucocorticoids, causing physiological changes evidenced by the communication between the immunological, endocrine and nervous systems. Acute stress harms the body, increasing the risk of chronic diseases such as cancer, cardiovascular diseases, diabetes, dementia, and depression, with a global impact on morbidities and mortality^(^ 28 ^)^.

It is worth noting that even though the participants identified physical and psychological changes amidst the SARS/CoV2/COVID-19 pandemic, they recognize that the social isolation measures and the closing of commercial businesses and borders are important to maintain the health of the general population, given the large circulation of people in the border area.

The contingency table shows that the retraction of the global economy has affected n the income of families, considering the inability of the job market to maintain job posts^(^ 7 ^)^. The effects of social isolation have already been experienced in the international border regions. The sectors addressed here manifested loss of family income, including among those respondents who have stable jobs. A potential explanation is that the family income in these cases is composed of the income of spouses and/or other family members working in economic activities that depend on the consumption of goods and services and circulation of goods.

In the south of the Brazilian border, the city of Foz do Iguaçu, PR (bordering Ciudad del Este, Paraguay and Puerto-Iguazu, Argentina) forms an international shopping and tourism hub, with economic influence and direct and indirect employability for the cities along the border, constituting the primary source of income for small businesses and families. Naturally, people in these economic sectors perceive the influence of border and businesses closure on family income. The largest number of people who answered that closing the border and businesses was not a useful measure to prevent illness caused by SARS/CoV2/COVID-19 belonged to these economic sectors.

Even though it is clear that the stay-at-home orders and the closure of borders and businesses will cause families’ financial problems, the respondents confirmed there was a need to adopt these sanitary measures. Nonetheless, the task of balancing the effects of social isolation on the economy and social determinants of health is complex^(^ 12 ^,^ 29 ^)^.

High-income countries rapidly allocated financial resources to support citizens and businesses as soon as they envisaged the economic threat posed by the COVID-19. The European Union and the USA announced the release of funds, based on stimulus bills, to protect people against economic impacts^(^ 29 ^)^. The Brazilian government acted in the same direction when it implemented economic measures to decrease the impact of the COVID-19 pandemic^(^ 14 ^)^.

However, the emerging countries’ financial resources are limited and the consequences of these measures might reverberate in the near future. Indicators revealed that emerging markets were the first from which investors fled. In a globalized economy, it implies limiting governments, businesses, and families’ access to credit, possibly restricting the health system budge in a time in which its capacity needs to be urgently expanded. The SARS/CoV2/COVID-19 pandemic is a threat to the world economy, hence, a threat to the financing of health systems^(^ 29 ^-^ 31 ^)^.

The workers from the sectors addressed in this study consider that health services are ready to meet the needs of the population amidst the COVID-19 pandemics. The respondents show a considerable degree of trust in health institutions. This level of confidence is probably due to information disseminated by the media concerning the public health measures adopted by the SUS; that there is still sufficient capacity to care for suspected cases and individuals infected with COVID-19; while there is no news about lack of beds or the existence of queues to schedule visits in the health services located in the border cities.

Those working in commercial/tourism businesses more frequently reported mood changes, sadness, and anxiety, compared to individuals working in other sectors. Note that physical and psychological complaints were very frequently reported by all the groups included in this study, revealing the impact of the pandemic and social isolation on people’s health, a finding that is in line with a study conducted in China^(^ 32 ^)^.

This study’s main limitation refers to the impossibility of establishing any causal relations. Nevertheless, the fact that data were collected during the period in which the international borders and commercial businesses were closed strengthens the answers provided. Another limitation is that this study includes only the population living in Brazil. That is, the populations living in the bordering countries, Paraguay and Argentina, were not heard even though a portion of these populations does seek healthcare services in Brazil.

As for the contributions to the advancement of scientific knowledge, the results presented here can support comparisons with future studies addressing the repercussions of sanitary measures to contain the spread of SARS/CoV2, on the health of the population and economy, especially in strategic international frontier regions.

## Conclusion

Data analysis revealed that social isolation and the closure of borders strongly affected the population’s health and the region’s economy. Repercussions in the individuals’ health include physical and psychological changes experienced by a statistically significant portion of the population addressed in this study. There were reports of depression, diseases, and physical pain not previously experienced. In economic terms, a decrease in consumption/sales was verified due to job loss, with a consequent decrease in family income. Those working in commercial/tourism businesses were the most frequently affected in terms of health and income and, were also resistant against the measures, though they understood the importance of implementing social isolation measures (e.g., closure of borders, commercial businesses, schools, and universities) to protect the population against SARS/CoV2. Additionally, they also manifested their trust in the health services’ capacity to face the COVID-19 pandemic, though there is a notion that SUS may become overburdened.

Regarding this study’s practical considerations, we highlight that a lack of bilateral agreements to regulate sanitary measures to control the pandemic in an integrated and solidary manner between countries sharing borders (not only geographical borders) constitutes a critical gap. The reason is that there is great population mobility due to family and friendship ties, in addition to education and labor relations, a search for health services, and other social devices on both sides of the border, inherent to the dynamics and social and economic interdependence between these supranational regions. Meanwhile, it is known that even though there is no support of bilateral sanitary agreements, unofficial acts make access more flexible to assist neighboring populations.

It is crucial to implement bilateral political agreements to deal with the public health of international border regions amidst the global emergence imposed by SARS/CoV2/COVID-19, considering that as COVID-19 is increasingly and unevenly disseminated in the population strata, those in greater social vulnerability may be left unassisted. By showing the perception from a portion of the residents of the border region regarding the consequences of the pandemic on the economy and the health of the population, this paper reveals the risk of social collapse, resulting from unassisted populations who will eventually seek in both sides of the border, legal or illegal ways to obtain subsistence or ensure survival, whether to access essential goods or health services.

## References

[1] World Health Organization (2020). Coronavirus (COVID-19).

[2] Wenzhong L, Hualan L (2020). Covid-19: attacks the 1-Beta chain of hemoglobin and captures the porphyrin to inhibit human heme metabolism. ChemRxiv.

[3] Xu Z, Shi L, Wang Y, Zhang J, Huang L, Zhang C (2020). Pathological findings of Covid-19 associated with acute respiratory distress syndrome. Lancet Respir Med.

[4] Joob B, Wiwanitkit V (2020). Covid-19 can present with a rash and be mistaken for dengue. J Am Acad Dermatol.

[5] Nussbaumer-Streit B, Mayr V, Dobrescu AI, Chapman A, Persad E, Klerings I (2020). Quarantine alone or in combination with other public health measures to control Covid-19: a rapid review. Cochrane Database Syst Rev.

[6] Vellingiri B, Jayaramayya K, Iyer M, Narayanasamy A, Govindasamy V, Giridharan B (2020). Covid-19: a promising cure for the global panic. Sci Total Environ.

[7] Organização das Nações Unidas (2020). Covid-19 destrói o equivalente a 14 milhões de empregos na América Latina e Caribe, diz OIT.

[8] Aikes S, Rizzotto MLF (2018). Integração regional em cidades gêmeas do Paraná, Brasil, no âmbito da saúde. Cad Saude Publica.

[9] Instituto Brasileiro de Geografia e Estatística (2020). Informações sobre saúde, abrangendo, demografia, infraestrutura e gestão da saúde, morbidade e causas de mortalidade.

[10] Wang Z, Tang K (2020). Combating Covid-19: health equity matters. Nat Med.

[11] Krishnakumar B, Rana S (2020). Covid-19 in India: Strategies to combat from combination threat of life and livelihood. J Microbiol Immunol Infect.

[12] Karnon J (2020). The case for a temporary Covid-19 income tax levy now, during the crisis. Appl Health Econ Health Policy.

[13] Ujvari SC (2012). Pandemias: a humanidade em risco.

[14] Governo Federal do Brasil (2020). Medidas econômicas voltadas para a redução dos impactos da Covid-19.

[15] Weston S, Frieman MB (2020). Covid-19: knowns, unknowns, and questions. Msphere.

[16] Gobierno Federal de Paraguay (2020). Poder Legislativo. Ley n° 6.524.

[17] Gobierno de la República Argentina (2020). Decreto de Necesidad y Urgencia (DNU) 260/2020.

[18] Secretaria do Estado do Paraná (2020). Decreto nº 4.230/2020. Dispõe sobre as medidas para enfrentamento da emergência de saúde pública de importância internacional decorrente do Coronavírus - COVID-19.

[19] Anderson RM, Heesterbeek H, Hollingsworth TD (2020). How will country-based mitigation measures influence the course of the Covid-19 epidemic. Lancet.

[20] Ministério da Saúde (BR) (2020). Coronavírus: veja lista completa de hospitais que serão referência no Brasil.

[21] Lorenz C, Azevedo TS, Chiaravalloti-Neto F (2020). Covid-19 and dengue fever: a dangerous combination for the health system in Brazil. Travel Med Infect Dis.

[22] Ribas RM (2020). Coronavirus Disease 2019 (COVID-19) and healthcare-associated infections: emerging and future challenges for public health in Brazil. Travel Med Infect Dis.

[23] Litewka SG, Heitman E (2020). Latin American healthcare systems in times of pandemic. Dev World Bioeth.

[24] Cheung YT, Chau PH, Yip PS (2008). A revisit on older adults' suicides and Severe Acute Respiratory Syndrome (SARS) epidemic in Hong Kong. Int J Geriatr Psychiatry.

[25] Mamun MA, Griffiths MD (2020). First Covid-19 suicide case in Bangladesh due to fear of Covid-19 and xenophobia: possible suicide prevention strategies. Asian J Psychiatr.

[26] Goyal K, Chauhan P, Chhikara K, Gupta P, Singh MP (2020). Fear of Covid 2019: first suicidal case in India. Asian J Psychiatr.

[27] Yao H, Chen JH, Xu YF (2020). Rethinking online mental health services in China during the Covid-19 epidemic. Asian J Psychiatr.

[28] Turner L, Galante J, Vainre M, Stochl J, Dufour G, Jones PB (2020). Immune dysregulation among students exposed to exam stress and its mitigation by mindfulness training: findings from an exploratory randomised trial. Sci Rep.

[29] Kentikelenis A, Gabor D, Ortiz I, Stubbs T, McKee M, Stuckler D (2020). Softening the blow of the pandemic: will the International Monetary Fund and World Bank make things worse?. Lancet Glob Health.

[30] Sousa GJB, Garces TS, Cestari VRF, Moreira TMM, Florencio RS, Pereira MLD (2020). Estimation and prediction of COVID-19 cases in Brazilian metropolises. Rev. Latino-Am. Enfermagem.

[31] Díaz-Narváez V, San-Martín-Roldán D, Calzadilla-Núñez A, San-Martín-Roldán P, Parody-Muñoz A, Robledo-Veloso G (2020). Which curve provides the best explanation of the growth in confirmed COVID-19 cases in Chile. Rev. Latino-Am. Enfermagem.

[32] Qiu J, Shen B, Zhao M, Wang Z, Xie B, Xu Y (2020). A nationwide survey of psychological distress among Chinese people in the Covid-19 epidemic: implications and policy recommendations. Gen Psychiatr.

